# Little impact of new mutations on mammalian trait variation

**DOI:** 10.1371/journal.pbio.3002825

**Published:** 2024-09-27

**Authors:** Beth L. Dumont

**Affiliations:** 1 The Jackson Laboratory, Bar Harbor, Maine, United States of America; 2 Graduate School of Biomedical Sciences, Tufts University, Boston, Massachusetts, United States of America; 3 Graduate School of Biomedical Science and Engineering, The University of Maine, Orono, Maine, United States of America

## Abstract

New mutations provide the source of all genetic variation but their impact on trait variation remains poorly understood. This Primer explores a new study published in PLOS Biology which addresses this question, finding that new mutations exert only weak effects on some traits in mice.

## Teaser

New mutations provide the source of all genetic variation but their impact on trait variation remains poorly understood. A new study published in *PLOS Biology* addresses this question, finding that new mutations exert only weak effects on some traits in mice.

Mutations are responsible for all genetic diversity, including variants associated with inherited diseases and adaptive traits. Historically, mutation rates were inferred indirectly by estimating the frequency of individuals with mutant phenotypes in populations or by comparing genetic differences between distantly related individuals or species. Modern high-throughput sequencing now enables the direct and comprehensive detection of new mutations by comparing parent and offspring genomes. Using this approach, recent studies have uncovered remarkable variability in mutation rates within and between genomes [1] and provided new insights into their mechanistic origins [2].

However, sequencing data alone cannot reveal the *effects* of new mutations. Even as fundamental knowledge of mutation rates and variability accumulates for diverse species, the phenotypic consequences of new mutations remain less well understood. This knowledge gap limits understanding of how traits evolve in nature, under artificial selection, and in settings when natural selection is weakened. This latter context carries special relevance to our own species. Recent medical and technological advances have reduced the ability of natural selection to weed out harmful mutations from human genomes. Thus, in an irony appreciated by geneticists for over 70 years, modern medicine has actually set humans on a path to steadily accumulate deleterious mutations, with a matched decline in overall fitness [3–5]. The rate of this fitness decline and the urgency of our self-inflicted “mutation problem” are governed by the average fitness effects of new mutations, emphasizing the fundamental importance of this parameter.

**Fig 1:**
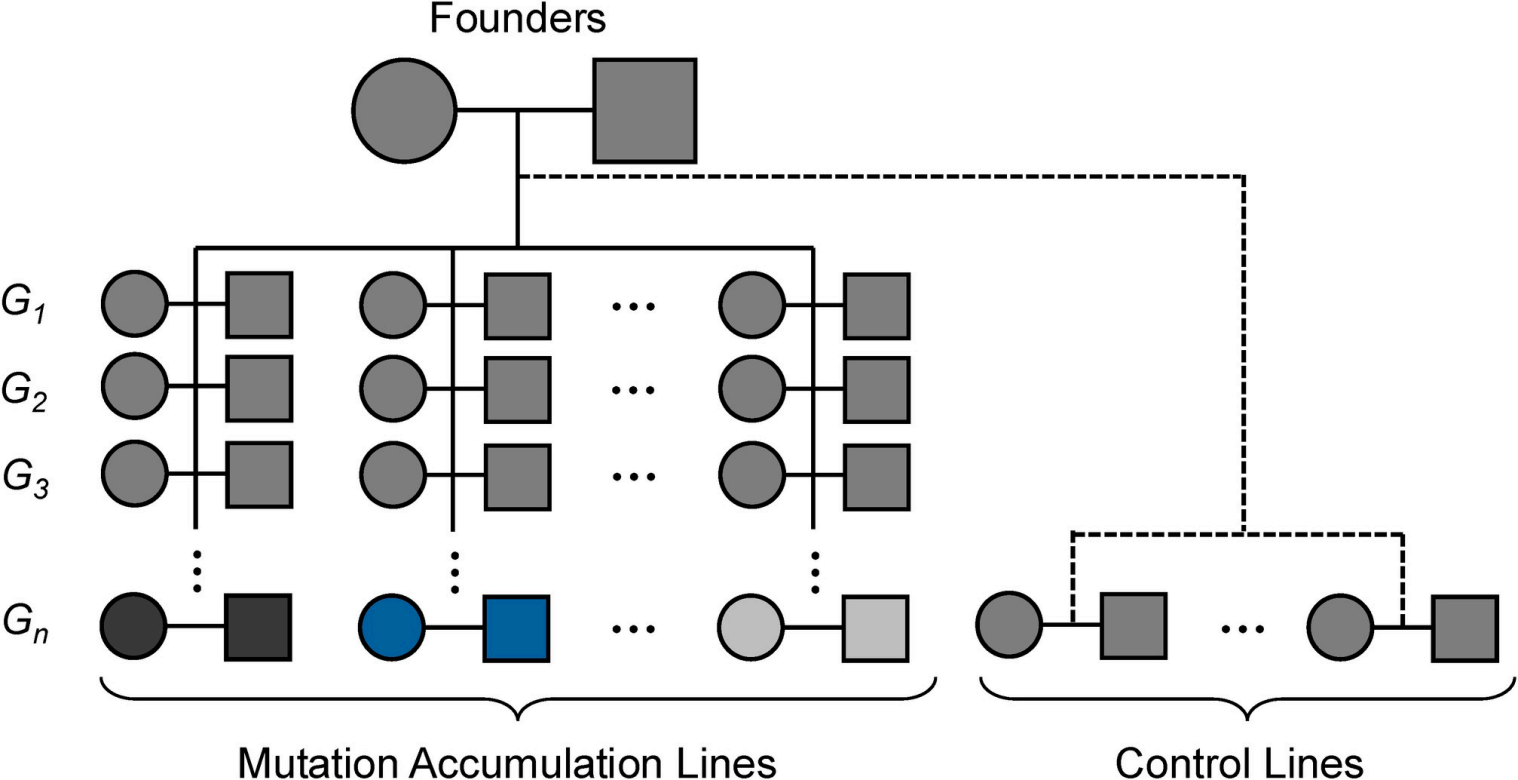
Schematic of a mutation accumulation experiment in a sexually reproducing species. Mutation accumulation (MA) provides an experimental framework for assessing mutation rates and the effects of new mutations on traits of interest. MA experiments typically initiate from a single founder pair of genetically identical individuals. This aspect of experimental design ensures that differences between replicate MA lines reflect the impacts of new mutations, rather than inherited variation. Independent MA lines are maintained for a large number of generations through strict sib-sib mating, minimizing the ability of natural selection to act on new mutations. With the exception of mutations associated with inviability or sterility, accumulated mutations therefore provide a read-out of the underlying spectrum of new mutations in a genome, independent of selection. Comparisons across replicate MA lines expose the variability of mutation accumulation across independent evolutionary trials and the amount of variation for surveyed traits that arises from new mutations at each generation. Equally important to the MA lines themselves are the control lines, which provide a baseline for the expected level of phenotype variation in the absence of accumulated mutations. Controls can be derived by freezing embryos from experimental founders and reviving them contemporaneously with the final generations of the MA experiment. Given the limited number of elapsed generations, any trait differences between experiment founders and control lines can be ascribed to factors other than new mutations, including changes in environment, epigenome, or microbiome. The schematic employs standard pedigree nomenclature, with circles indicating females and squares denoting males. The accumulation of independent mutations along each example lineage introduces phenotypic variation across lines, as illustrated by different fill colors.

Mutation accumulation (MA) experiments provide a powerful framework for estimating the phenotypic effects of new mutations [6]. In a typical MA experiment, isogenic or inbred organisms are bred over many generations under conditions that minimize natural selection (**Fig 1**). By tracking changes in trait values over time, researchers can assess the impact of new mutations on trait variation, including fitness-related phenotypes.

The first MA experiment was performed nearly a century ago in *Drosophila melanogaster* [7]. Numerous MA experiments have since been conducted in single-cell organisms, plants, and non-vertebrate animals [6], reflecting the ease of maintaining large numbers of replicate MA lines for small organisms with short generation times. However, differences in DNA repair mechanisms and life history between vertebrates and other organisms underscore the need for additional MA studies, particularly in species that may offer insights into human mutation accumulation.

In this issue of *PLOS Biology*, Chebib and colleagues tackle this important challenge [8]. The authors present findings from the largest MA experiment ever conducted in a mammalian species, featuring 55 independent MA lines derived from a single inbred founder pair of mice and maintained via strict sib-sib mating for >20 generations. Mice were assessed for morphological (body weight and tail length) and fitness-related (offspring survival and litter size) traits to monitor trait changes over time. Chebib and colleagues’ study represents a true tour de force, spanning 7 years of dedicated breeding, meticulous colony record management, and routine phenotyping.

On average, the authors observed that morphological trait values declined, whereas litter size and survival increased over the experiment. These phenotypic changes may result from (i) accumulated mutations; (ii) non-genetic factors such as changes in the environment, microbiome, or epigenome; or (iii) a combination of both genetic and non-genetic factors. To tease apart these possible explanations, Chebib and colleagues used sperm and oocytes collected from the founder animals to generate a bank of frozen embryos via in vitro fertilization (IVF). These cryopreserved embryos act as a time-frozen control group, preserving the founder genomes in an arrested state. Cryopreserved embryos were revived when the MA lines reached 16 generations, briefly maintained through strict sib-sib mating, and phenotyped for the same traits as the MA lines. Due to the limited number of accumulated mutations, phenotypic differences between the founders and control lines must largely stem from non-genetic factors.

Interestingly, the morphological trait differences observed in the control lines were only slightly less than those observed in the MA lines, indicating that morphological trait evolution over the MA experiment was dominated by non-genetic contributors, not new mutations. Similarly, the increase in fitness-related traits over the MA experiment is fully explained by environmental changes. Mice were housed under constant and controlled conditions for the 7-year duration of the experiment, implying that even imperceptible environmental perturbations can lead to significant phenotypic changes.

Although most phenotypic evolution observed in the authors’ MA lines is attributable to nebulous non-genetic factors, Chebib and colleagues do find that new mutations modestly contribute to heritable variation in their 2 assessed morphological traits. This finding carries important implications for targeted breeding efforts in agricultural species, suggesting that new mutations may make small, albeit significant, contributions to the phenotypic response to artificial selection.

Chebib and colleagues’ study emphasizes several outstanding questions and opportunities for further investigation. First, their investigations monitored changes in a select number of morphological and life history traits. The heroic nature of this undertaking is not to be doubted, but the natural question emerges as to whether new mutations differentially contribute to variation for different traits. Indeed, both their work and earlier studies suggest lower mutational variation for fitness-related traits compared to morphological traits. Second, some studies have suggested that IVF-derived embryos may have increased mutation loads (e.g., [9]), raising the question as to whether cryopreservation truly halts mutation accumulation. Finally, the authors develop their MA lines from a single inbred strain. The presence of mutator alleles within populations [10] indicates that the phenotypic effects of new mutations could vary with genetic background, a prospect that could be addressed in future MA studies using different inbred strains.

Chebib and colleagues’ work marks a significant advance in our understanding of the phenotypic consequences of new mutations in mammals and provides the hard data needed to quantify the threat new mutations pose to the fate of our species. Based on the authors’ findings and assuming similarity of mutational properties in mice and humans, it seems unlikely that humans will confront a significant fitness reduction due to new mutations in the near-term future, echoing earlier conclusions [4]. Instead, Chebib and colleagues assert that any threat to human civilization posed by an increasing mutation load is overshadowed by the more immediate dangers of climate change, overpopulation, and terrorism.
